# American tegumentary leishmaniasis diagnosis using *L. (V.) braziliensis* fixed promastigotes: a comparative performance of serological tests and spontaneous cure identification

**DOI:** 10.1186/s12879-019-4642-7

**Published:** 2019-11-29

**Authors:** Andresa Pereira Oliveira Mendes, Beatriz Coutinho Oliveira, Allana Maria S. Pereira, Maria Carolina Accioly Brelaz Castro, Marina Assis Souza, Maria Edileuza Felinto Brito, Fernanda Fortes Araújo, Andrea Teixeira-Carvalho, Olindo Assis Martins-Filho, Valeria Rêgo Alves Pereira

**Affiliations:** 1 Departamento de Imunologia, Instituto Aggeu Magalhães, FIOCRUZ, Av. Moraes Rego s/n, Cidade Universitária, Recife, Pernambuco CEP: 50670-420 Brazil; 2 Universidade Federal de Pernambuco, Programa de Pós-graduação em Inovação Terapêutica, Recife, Pernambuco Brazil; 3 Universidade Federal de Pernambuco, Laboratório de Parasitologia, Centro Acadêmico de Vitória, Recife, Pernambuco Brazil; 4 Instituto René Rachou, FIOCRUZ, Belo Horizonte, Minas Gerais Brazil

**Keywords:** ATL, *L. (V.) braziliensis*, Flow cytometry, ELISA, Cure criterion and spontaneous cure

## Abstract

**Background:**

The present study aimed to demonstrate the applicability of a flow cytometry-based serology approach to identify spontaneous cure by the detection of immunoglobulin G, and also, the diagnosis and cure criterion by the IgG1 isotype in American Tegumentary Leishmaniasis – ATL caused by *L. (V.) braziliensis*. Also, a comparison between flow cytometry with the serological conventional technique was performed.

**Methods:**

Forty five individuals were included in study. They were assessed in two moments: First, 8 subjects spontaneously cured of ATL, 8 healthy individuals and 15 patients who had a positive diagnosis for ATL were selected before treatment to identify spontaneous cure by immunoglobulin G detection. Secondly, 14 patients who were positive for ATL were selected and had their blood collected before and 1, 2 and 5 years after treatment, respectively, for the diagnostic tests (ELISA and flow cytometry) and cure criterion evaluation using the IgG1 isotype.

**Results:**

The analysis of the mean percentage of positive fluorescent parasites (PPFP) along with the titration curves of IgG anti-fixed promastigotes of *L.(V.)braziliensis*, confirmed the applicability of this method for monitoring spontaneous cure in ATL with outstanding co-positivity (100%) and co-negativity (100%) performance indexes. Regarding the results of the comparison between flow cytometry and ELISA it was seen that there was a better accuracy of the first one in relation to the other. When IgG1 applicability was evaluated, it was observed that before treatment, 36.8% of the patients were negative; in patients 1 year post-treatment, 82.3%; 2 years post-treatment, 27.2% and in patients 5 years post-treatment, 87.5%. The overall analysis of the results suggests that flow cytometry can be applied to ATL detection, and that the use of IgG1 isotype has possibilities to contribute as a more specific diagnostic method.

**Conclusions:**

Therefore, this area has great perspectives use for the diagnosis and cure criterion, and also it can be scaled up with the possibility to characterize the different clinical stages of the disease. Together, these findings demonstrate the applicability of a flow cytometry-based serology approach and opens up new avenues of research with this technique, such as the understanding the humoral response in ATL patients.

## Background

American Tegumentary Leishmaniasis (ATL) is an infectious, chronic, non-contagious disease which affects millions of people worldwide and is still a serious public health issue. The 3 most common species in Brazil are *Leishmania (Viannia) braziliensis, Leishmania (Viannia) guyanensis, and Leishmania (Leishmania) amazonensis*, respectively. ATL presents a variety of clinical forms ranging from limited cutaneous lesions until mucocutaneous disfiguration, and its evolution depends on the immunological status of the patient and the involved *Leishmania* species, where *Leishmania (Viannia) braziliensis* stands out as the main cause of ATL in Pernambuco [1–4].

A timely and adequate treatment is necessary to prevent disease aggravation to destructive and severe forms. The current treatment options present high toxicity and important adverse effects, none of which are sufficiently effective [5–7]. Besides, there is no cure criterion available based on the spontaneous cure assessment, what suggests that the patients who were spontaneously cured have developed an immune response capable of controlling their *Leishmania* infection [8, 9]. Due to that, the laboratorial diagnosis of ATL can be considered a challenge nowadays. Therefore, the search for new diagnostic tools is highly necessary. Although a variety of tests including serological, parasitological and molecular methods are available, the diagnosis of ATL is still unsatisfactory [10]. Serological methods based on antibody detection are the most widely used tests worldwide [11–13], however, a variable efficacy can be observed, since they might present low or no levels of *Leishmani*a-specific antibodies, those of which can still remain positive for years. The cross-reactivity with other diseases, including Chagas disease and visceral leishmaniasis are also one of their biggest limitations [6, 14]. This stimulates the search for novel methodologies that can reach a higher efficacy.

Up to the moment, ATL’s diagnosis is performed by an association of epidemiological, clinical and laboratorial results. Since there is no diagnostic test considered as the gold standard for ATL, the association of these elements is necessary to achieve the final diagnosis [6, 15]. The parasitological test is made through smear (parasite direct search), culture in specific medium or histopathological tests. The smear has a sensitivity of 50 to 70% and it depends on the number of parasites on the slide. The test’s positivity is inversely proportional to the evolution time of the cutaneous lesion, being lower after 1 year. Although it is a simple, quick and inexpensive technique, it is not able to detect parasites in every patient. The sensitivity of isolation in culture is generally low, around 20 to 40%, and in many cases, it is related to the scarcity parasites in the lesions, especially when it comes to *L. (V.) braziliensis* [16–19].

Immunological methods such as the Montenegro’s skin test (IDRM) rely on the evaluation of the patient’s cellular immune response, whereas indirect immunofluorescence (IFI), immunoenzymatic assay (ELISA) and western blot are based on the humoral response, the latter being more commonly used. In ATL, the immunological procedures are the only applied methods which can detect the disease in its initial stages before the beginning of the treatment [2, 20]. The IDRM has been used as an important resource in the immunological diagnosis of ATL given its great sensitivity and specificity. Although it shows a positive result in most cases of ATL (90%), the result is negative in recent lesions, in the diffuse cutaneous form and in immunosuppressed patients. In endemic areas, the test is usually positive due to subclinical infections. In addition, the test does not differentiate infection from disease or an active disease from a previous one [16, 19, 21]. IFI, ELISA and Western blot present important disadvantages especially regarding sensitivity, specificity and poor reproducibility. Apart from that, they may cross-react with other trypanosomatids. It is also known that low levels of antibodies are detected by these techniques, and that there is no correlation between circulating antibodies with the presence of an active infection [22, 23]. Because of that, diagnostic methods are urgently needed and researchers around the world have been developing new technologies to ensure the continuous improvement of the available tools [23–25].

The first flow cytometer was a single-parameter instrument which could only detect the size of the cells. Currently, highly sophisticated instruments with the ability of detecting 14 parameters simultaneously have become a reality [23]. This tool has made a revolution in the diagnosis field since it could enable a precise evaluation of multiple biological processes. Although one might raise the limitations associated with its cost, it must be recognized that flow cytometers are already well-established in several reference laboratories, including hospitals and clinics that diagnose patients with HIV [24], and also that its sensitivity is usually higher when compared to other serological tests. Therefore, flow cytometry arises as an extremely versatile technology, associating functionality and precision. It is used in several laboratorial investigations including molecular biology, pathology and immunology, with a vast application in healthcare, especially in transplants, hematology, immune system evaluation, tumor immunology and chemotherapy [23, 25–28]. Several efforts have been made to develop reliable flow cytometry serological approaches for both ATL’s diagnosis and cure monitoring, the main one by using distinct antigen preparations to detect anti-*Leishmania* antibodies [29, 30]. Together, these approaches have demonstrated that flow cytometry-based methods can be applied to the diagnosis and post-therapeutic cure assessment in ATL.

In order to improve and innovate flow cytometry assays, this work intended to use anti-fixed *Leishmania (Viannia) braziliensis* promastigote IgG antibodies to demonstrate its applicability in identifying ATL spontaneous cure by differential reactivity when compared to patients with active infection, and also, the use of the IgG1 isotype for the diagnosis and cure criterion, comparing it with the conventional serological methods for ATL.

## Methods

### Study population

**Fig. 1:**
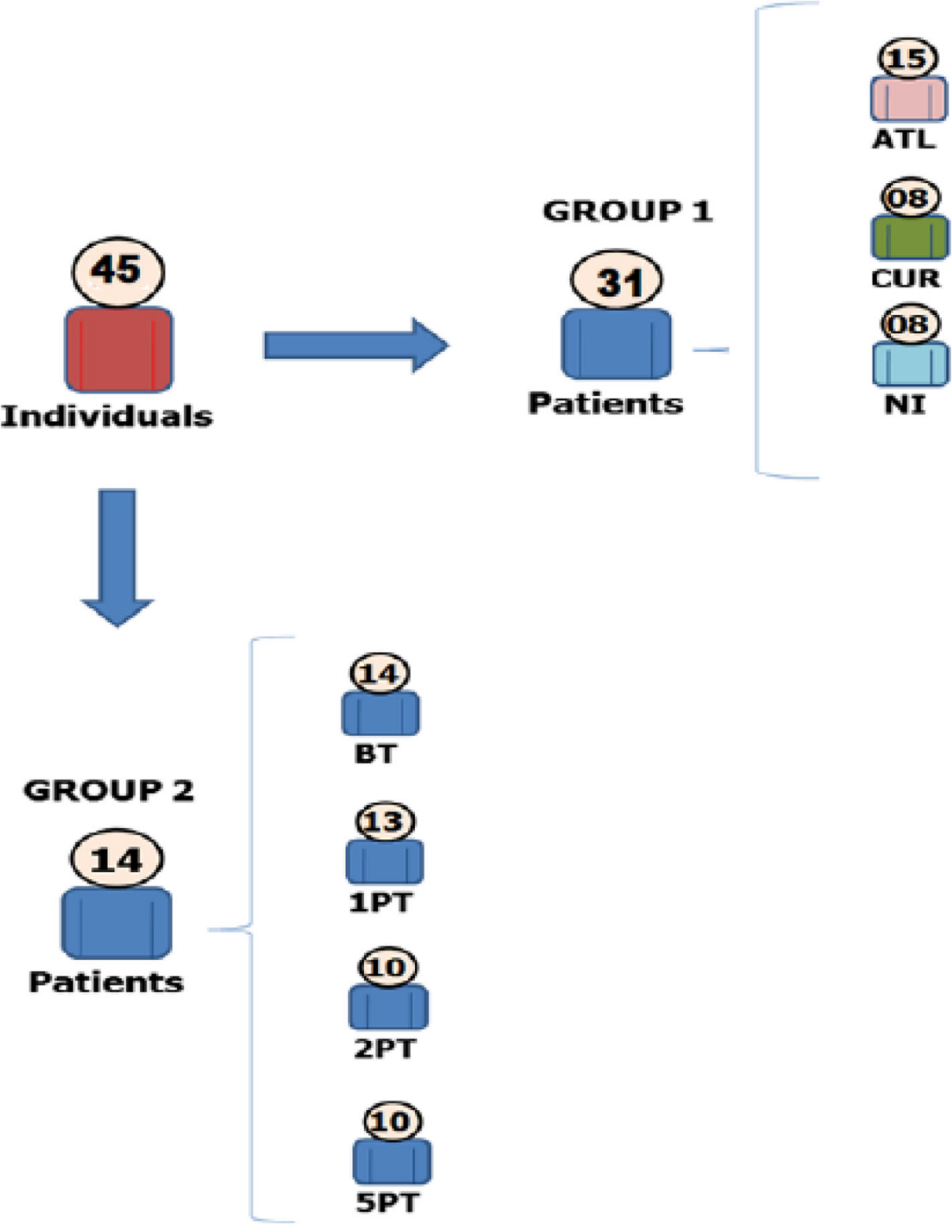
Design of the study population. Group 1 consisted of 31 individuals, splitted in: patients with American Tegumentary Leishmaniasis (ATL *n* = 15), non-infected individuals (NI *n* = 8), spontaneously cured patients (CUR n = 8). Group 2 was formed by patients with ATL before treatment (BT *n* = 14). Of these, 13, 10 and 10 individuals performed a new blood collection 1, 2 and 5 years, respectively, post therapeutic (PT) intervention and complete healing of the lesions

Forty five individuals participated in this study and they were only included when they had their positivity confirmed in at least two tests, including: Montenegro skin test, indirect immunofluorescence and PCR (Fig. 1). Twenty nine patients with positive diagnosis (ATL) were selected before treatment. The experimental design was carried out in two stages: In the first moment, 15 patients with positive diagnosis for ATL were selected before treatment. Subjects spontaneously cured ATL (CUR = 08) showed complete healing of their lesions without any therapeutic intervention. Serum samples from 8 healthy individuals with no documented infection or exposure to *Leishmania* parasites were included as non-infected controls (NI). In the second moment, 14 patients with positive diagnosis for ATL were selected. They had active lesions and no concomitant cutaneous diseases. Of these, 13, 10 and 10 individuals performed a new blood collection 1, 2 and 5 years, respectively, after treatment and healing of the lesions. All patients with positive ATL came from endemic areas in Pernambuco State. All samples were evaluated as for clinical, epidemiological and laboratorial criteria and all individuals agreed and signed the “Term of Free and Informed Consent”. CPqAM/Fiocruz (Protocol no. 001300950000–7). The Research Ethics Committee has approved the experimental protocols.

### Parasite preparation

*L. (V.) braziliensis* promastigotes (MHOM/BR/75/M2903) were cultivated in Schneider’s medium until they reached exponential phase. The parasites were centrifuged at low-speed (100×g), 25 °C for 10 min and were recovered from the supernatant after 10 min of rest at room temperature. The supernatant was transferred to another tube and washed 3 times in saline buffer pH 7.2, containing 10% heat-inactivated fetal bovine serum (phosphate buffered saline (PBS)-10% FBS) at 4 °C, 871×g for 10 min. The pellet of parasites was thoroughly homogenized, re-suspended and fixed in an equal volume of 1% paraformaldehyde, being incubated overnight. The parasites were then washed with PBS-10% FBS, re-suspended and counted using Neubauer chamber, so the concentration was adjusted for the assays.

### Flow Cytometry acquisition and analysis

Flow cytometry acquisition and analysis for IgG detection of anti-fixed *L. (V.) braziliensis* promastigotes were performed according to Oliveira et al. (2013). Briefly, the parasite suspension (2.5 × 10^5^/well) was incubated in 96-well U-bottom plates at 37 °C for 30 min in the presence of serial dilutions of inactivated serum samples (from 1:64 to 1:8192). After the incubation, the parasites were washed twice with 150 μl of PBS-10% FBS (1000×g for 10 min at 4 °C) and re-incubated at 37 °C for 30 min in the dark with anti-human IgG antibody conjugated with fluorescein isothiocyanate – FITC (Sigma Chemical Corp., St. Louis,MO) previously diluted 1:4000 in PBS – 10% FBS. The FITC-labeled parasites were washed twice and fixed with FACS fix solution for 30 min and stored at 4 °C up to 24 h before flow cytometry analyses.

For IgG1 acquisition and analysis, the assay was performed according to Rocha et al. (2002). A parasite suspension (5 × 10^6^/well) was incubated in 96-well U-bottom plates for 30 min at 37 °C with different patients’ serum dilutions (1:64 to 1:8192). After this period, the plates were washed twice with 150 μl of PBS-10% FBS (800×g for 10 min, 4 °C). Fifty microliter of human anti-IgG subclasses conjugated to biotin (Sigma ChemicalCorp., St. Louis, MO), were added and the plates were incubated at 37 °C for 30 min. Then, they were washed twice with PBS-10% FBS. Afterwards, the plates were again stored at 37 °C for 30 min in the presence of 10 μL of streptavidin conjugated to phycoerythrin (Gibco), diluted at 1:100 in PBS-10% FBS. After this period, the plates were washed and fixed with 200 μL of fixing solution. The samples were stored for at least 30 min at 4 °C until flow cytometry acquisition. Flow cytometric acquisition (20,000 events per sample) was performed on a FACSCalibur cytometer (Becton Dickinson) and the “CellQuest Pro” software was used for both data storage and analysis. The parasites were identified based on their specific forward (FSC) and side (SSC) light-scattering properties. Parasites were selected by gating on the FSC × SSC dot-plot distribution. The relative FITC fluorescence intensity for each parasite was analyzed by a single histogram representation. A marker was set on the internal control to confine the nonspecific binding of FITC-conjugated antibody up to 2% and used to determine for each sample the percentage of positive fluorescent parasites (PPFP) as previously described by Oliveira et al. (2013).

### Enzyme-linked Immunosorbent assay - ELISA

The assay was performed following a protocol developed at the Immunogenetics Laboratory of Aggeu Magalhães Institute (FIOCRUZ/PE – Recife – Brazil). The plate was sensitized with 100 μL of the antigen (5 μg/mL) per well, previously diluted in Carbonate/Bicarbonate buffer 0.06 M pH 9.6. The plate was incubated at 4 °C/ 18 h in a humid chamber. After this period, the plates were washed 3 times with PBS 0.015 M pH 7.2 containing 0.05% of tween 20. On the first wash, the supernatant was discarded and on the other 2, the plates stayed with the washing solution for 10 min before discarding. Sera were diluted from 1:40 to 1:1280 in PBS 0.015 M pH 7.2 containing 0.05% of tween 20 and powdered milk. To each plate it was added a negative standard with the same dilutions of the samples, so the mean and standard deviation could be calculated. One hundred microliter of the diluted sera was added per well and the plate was stored at 37 °C for 1 h. The plates were washed again like previously mentioned. One hundred microliter per well of the anti-human IgG (gamma chain specific) conjugated to peroxidase was added on the ideal titer for the test (Calbioquem, 1:2500). Then, the plate was washed as described. One hundred microliter per well of the chromogen (5-amino salicylic acid/OPD) was added. After 1 h of incubation in humid chamber at room temperature, the reaction was quenched with 25 μL per well of NaOH 1 M and the analysis was made in a spectrophotometer at 450 nm wave length. For results interpretation, the reactivity limit of each positive sera was calculated based on the absorbance mean of the negative standard dilutions plus 2 times the standard deviation. Those considered positive were the samples with had higher titers than the result of the calculi described above.

### Statistical analysis

Performance indexes were calculated to determine flow cytometry’s applicability in diagnosing ATL and assessing spontaneous cure, including “co-positivity” or sensitivity = ([true positive / real positive] × 100) and “co-negativity” or specificity = ([true negative / real negative] × 100). In addition, data were submitted to the ROC curve analysis to identify the area under the curve (AUC) as the global test’s accuracy. The data set was also analyzed by the two-graph receiver operating characteristic (TG-ROC) and Likelihood ratios - LR (LR+ = Se / (1–Sp) and LR- = (1–Se) / Sp).

Performance indexes of ELISA and flow cytometry can also be evaluated by classifying the results in four categories according to the presence of lesion (group before treatment - BT) or absence of lesion (group post treatment – PT). These categories were defined as: true positive = presence of lesion and positive test; false-positive = absence of lesion and positive test; false-negative = presence of lesion and negative test and true-negative = absence of lesion and negative test (Table 1). The sensitivity was calculated by the ratio a/(a + c) translating thereby the proportion of patients with ATL (with lesion), that is to say, the proportion of ATL patients who were positive for ELISA or flow cytometry. Regarding specificity, it is related to the proportion of individuals without the clinical manifestations of ATL (no lesion), meaning those who are negative can be determined by the ratio d/(b + d).

**Table 1:**
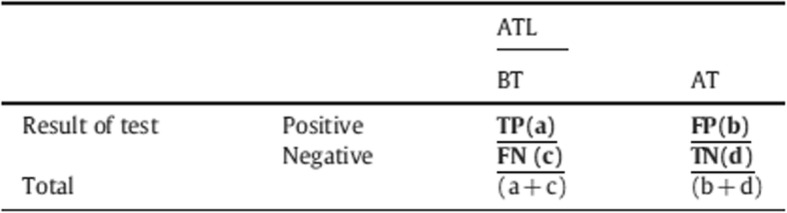
Categories for the classification of the patients from the test performance indexes

Statistical analyses were performed using the MedCalc® statistical software and the Computer Method for Diagnostic Tests – CMDT, version 1.0β (Berlin: Freie Universität Berlin; 1997. Copyright© 1997–1999 Jens Briesofsky).

## Results

### Applicability and performance indexes of flow cytometry for spontaneous cure assessment and monitoring

**Fig. 2:**
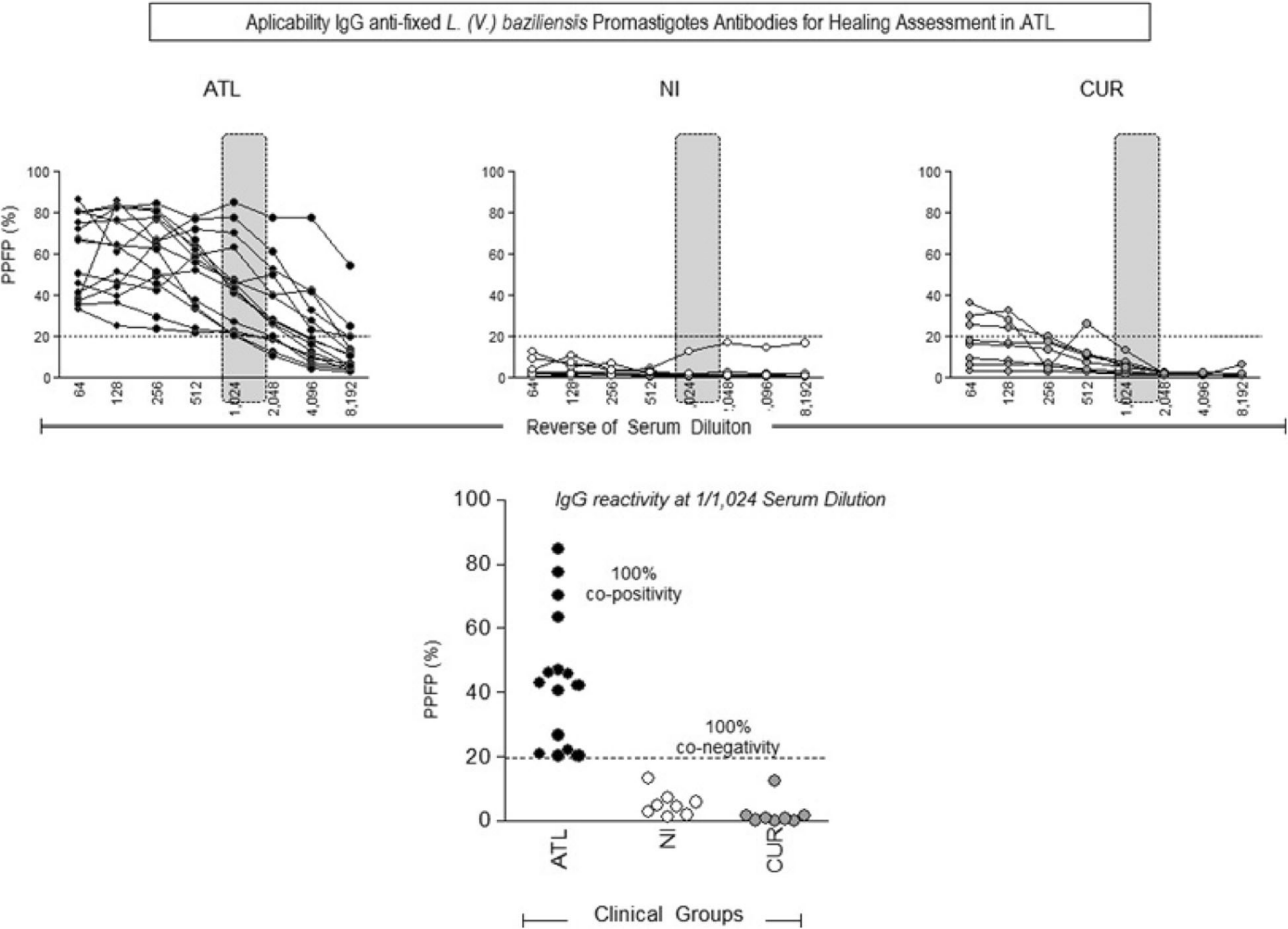
Titration curve of IgG antibodies anti-fixed *Leishmania braziliensis* promastigotes from the sera of patients with American tegumentary leishmaniasis (ATL ● n = 15); non-infected individuals (NI ○ n = 8) and spontaneously cured patients (CUR ● n = 8) (**a**). IgG antibodies anti-fixed *Leishmania braziliensis* from serum samples of ATL, NI and CUR groups at the 1/1024 dilution (**b**). The results were expressed as percentage of positive fluorescent parasites (PPFP). The dotted line represents the cut-off point between negative and positive results (cut-off = 20%)

Serum samples from 15 patients who were positive for ATL; 8 spontaneously cured (CUR) and 8 healthy individuals (NI) were tested by flow cytometry. The analysis of the mean PPFP values along with the titration curves of anti-fixed promastigote *Leishmania* IgG (from 1:64 to 1:8192) were used to select the 1:1024 serum dilution as previously reported by Pereira et al., 2012 and Oliveira et al. 2013. This serum dilution data also confirmed the higher segregation range among ATL, CUR and NI group (Fig. 2a). The Fig. 2b showed the co-positivity and co-negativity performance indexes confirming the selection of 1:1024 sera dilution as the most promising condition to discriminate the PPFP values of ATL from those provided by CUR and NI sera samples using the PPFP = 20% as the cutoff point. These results suggest the applicability of the method for monitoring spontaneous cure of the disease.

**Fig. 3:**
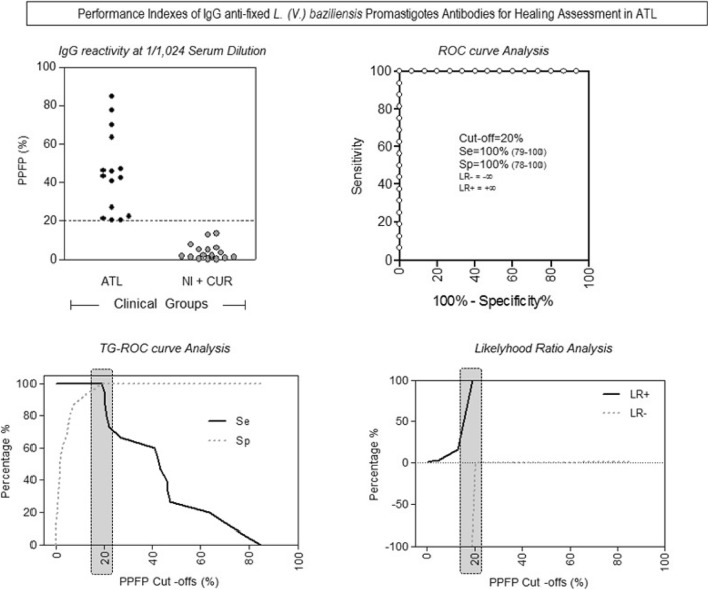
IgG antibodies anti-fixed *Leishmania braziliensis* from serum samples of ATL, NI + CUR groups at the 1/1024 dilution (**a**). Confirmation of the cut-off point by the ROC curve analyses, demonstrating the outstanding performance indexes (Sensitivity-Se; Specificity-Sp; Area under the curve-AUC **(b,c)**; and Positive/Negative Likelihood Ratio-LR+/LR- for ATL **(d)**. The results were expressed as percentage of positive fluorescent parasites (PPFP). The dotted line and grey bars represents the cut-off point between negative and positive results (cut-off = 20%)

ROC curves were constructed to confirm the selection of 1:1024 sera dilution as the most capable condition to discriminate the PPFP values among the groups (Fig. 3a) as well as to assess the performance of IgG anti-fixed *L. (V.) braziliensis* promastigotes antibodies for spontaneous cure assessment in ATL. The analysis of the area under the curve demonstrated an outstanding performance of the test (AUC = 1.0; IC95%:1.0–1.0). The performance analysis of IgG anti-fixed *L. (V.) braziliensis* promastigote antibodies for spontaneous cure assessment in ATL demonstrated the outstanding performance indexes with 100% of sensitivity (IC95% =79–100) and 100% of specificity (95%IC = 78–100) using the PPFP = 20% as the cutoff (Fig. 3b,c). Likelihood ratio (LR) corroborate the performance of IgG anti-fixed *L. (V.) braziliensis* promastigotes antibodies for spontaneous cure assessment in ATL, demonstrating that positive results (PPFP> 20%) has infinite times more chances to come from an ATL patient then NI + CUR individuals. On the other hand, negative results (PPFP< 20%) are unlikely to belong to an ATL individual, strongly suggesting the spontaneous cure (Fig. 3d).

### Applicability of flow cytometry for diagnosis in ATL

**Fig. 4:**
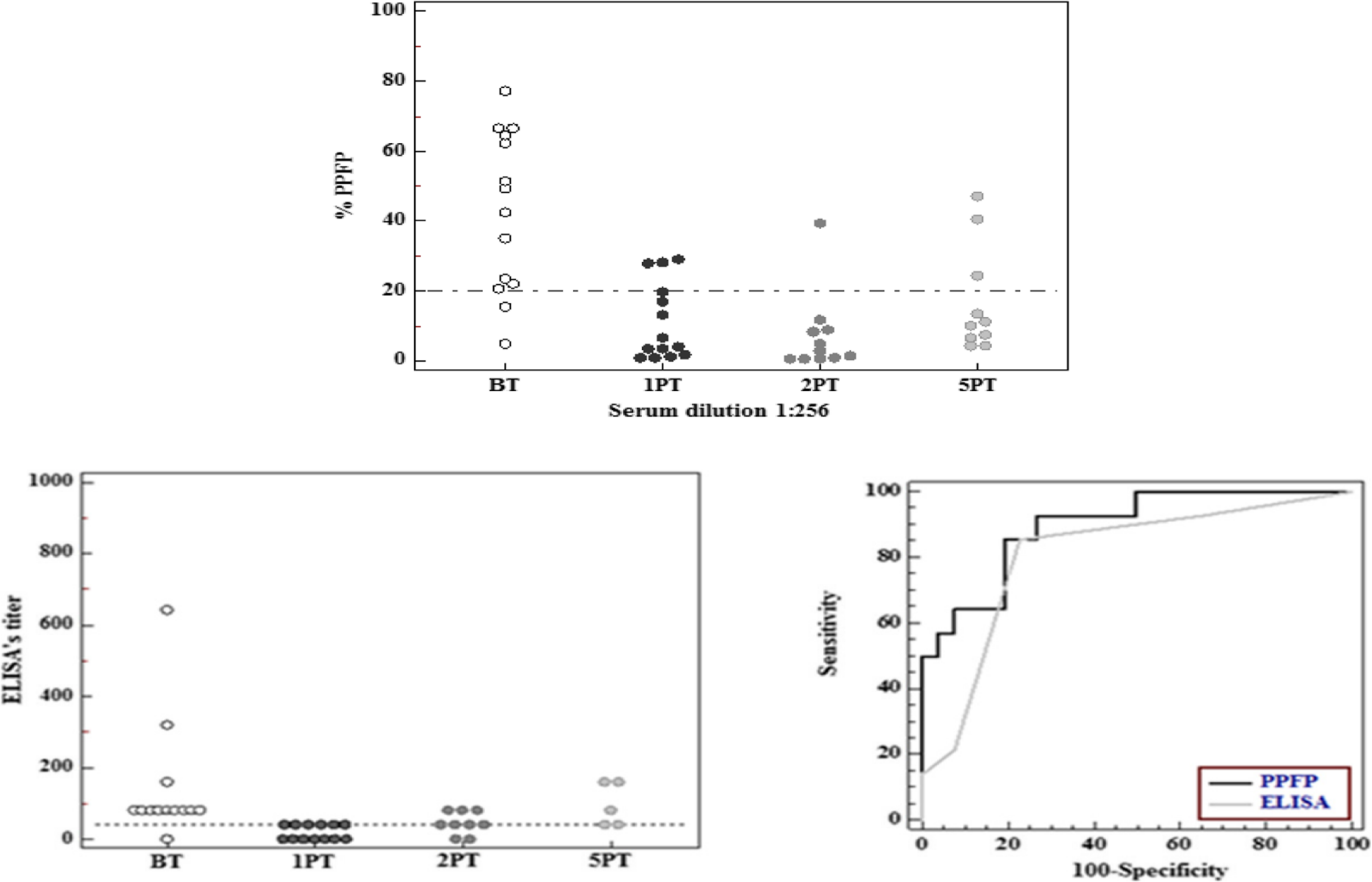
Applicability of flow cytometry in detecting patients with active ATL. The assay was performed using sera (diluted at 1/256) from patients BT, 1, 2 and 5 years PT. The results were expressed as percentage of positive fluorescent parasites (PPFP). The dotted line represents the cut-off point between negative and positive results (cut-off = 20%) **(a)**. IgG reactivity in sera from patients BT, 1, 2 and 5 years PT submitted to the ELISA test. The dotted line represents the cut-off point between negative and positive results (cut-off = 1:40) **(b)**. Comparison between the ROC curves of ELISA and Flow Cytometry **(c)**

In order to compare ELISA with flow cytometry for ATL’s diagnosis, tests evaluating IgG anti-promastigote forms of *Leishmania (Viannia) braziliensis* were performed in 14 patients before, and: 13 patients one; 10 patients two and 5 years after treatment. Using a 20% of PPFP cutoff, it was shown that it can be used to identify cutaneous cases of ATL, since it was possible to confirm 86% (12/14) of positivity in patients before treatment; and 77% (10/13), 80% (8/10) and 70% (7/10) of negativity, respectively, in patients one, two and 5 years after treatment (Fig. 4a).

Serial dilutions of the patients’ sera were added to the plates ranging from 1:40 to 1:1280. The patients who presented serum titers from 1:40 were considered positive for the ELISA test. From the evaluated sera, 92.8% (13/14) of the patients before treatment with Glucantime® were positive; 53.8% (7/13) were positive 1 year after treatment; 88.8% (8/9) were positive 2 years after treatment and 100% (5/5) of the patients 5 years after treatment remained positive for ATL in this test. To evaluate ELISA’s applicability in identifying active ATL, values of the dilution’s titer in which the patients were considered positive were plotted into a graph using the software MedCalc Statistical. It was possible to evaluate IgG reactivity in patients’ sera before and after treatment, as observed on the Fig. 4b. The chosen serum titers of ELISA and flow cytometry were used for the ROC curves (Fig. 4c). Based on the analysis of the ROC curve, it was possible to observe that ELISA’s AUC (ASC = 0.808; IC_95%_ = 0.652–0.915) was lower than flow cytometry’s (ASC = 0.896; IC_95%_ = 0.758–0.970). Thus, we can assure that flow cytometry had a better performance with a superior accuracy.

**Fig. 5:**
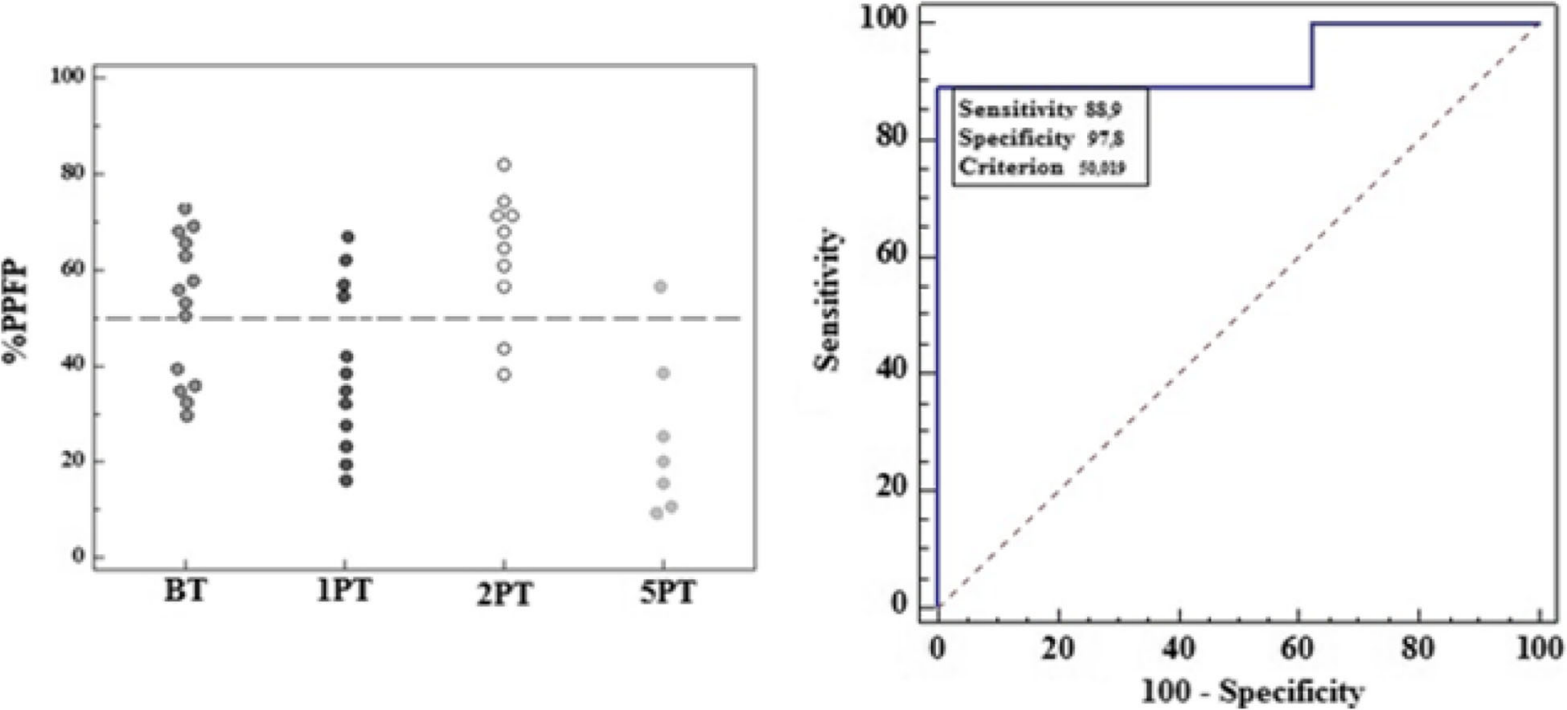
Applicability of flow cytometry in using IgG1 isotype to identify patients with active ATL **(a)**. Flow Cytometry’s ROC curve for the IgG1 isotype constructed with data from sensitivity and 100 – specificity. The area under the curve demonstrates the outstanding performance of the test (AUC = 0.931) **(b)**

After identifying the reactivity region and the cutoff, the confirmation of the test’s applicability was performed by detecting positive and negative patients. It was observed that in patients BT, 63.2% were positive; in patients 1 year after treatment, 17.7%; 2 years, 72.8% and in patients 5 years after treatment, 12.5%. These results can be observed in Fig. 5a. The values of the titer results from flow cytometry in the dilution 1:256 of the control sera were used to construct the ROC curve to evaluate the test’s accuracy (Fig. 5b). With the ROC curve analysis, it was observed that the AUC of flow cytometry was = 0.931; IC_95%_ = 0.698–0.997 showing that the test had an excellent performance.

## Discussion

The present work evaluated the detection of IgG antibodies anti-fixed *L. (V.) braziliensis* promastigotes by flow cytometry to identify active infection and spontaneous cure. We also suggested the use of the IgG1 isotype as an alternative tool for diagnosis and cure criterion of ATL. Several serological methods are able to diagnose and monitor ATL clinical cure by assessing the decrease of the parasite load after the complete healing of the lesions [31, 32]. Previously, Romero et al. (2005) demonstrated a decrease in reactivity levels of IgG after specific treatment, showing that ELISA has a better performance when compared to the IFA test. However, the cure criterions for ATL remain controversial, since the persistence of positivity in conventional serological tests can be observed after spontaneous or therapeutic cure.

Several efforts have been made aiming to build up a more reliable serological approach by using distinct antigen preparations to detect anti-*Leishmania* antibodies [33–35]. The spontaneously cured patients (CUR) are the target of immunological research possibly because they present a differential and more efficient protective response when compared to the cases of active ATL infection [9, 36]. Our results from CUR group showed a high performance of the method, presenting low reactivity of PPFP (PPFP< 20%) similar with NI individuals. The infection by *L. (V.) braziliensis* tends to be difficult to heal and sometimes it can evolve to the mucosal form. However, the spontaneous cure of the lesions suggests the development of an immune response capable of controlling the infection by *Leishmania* [9]. Previous studies showed that spontaneously cured patients present low levels of IgG antibodies, and that correlates to the decrease of the parasite load. Thus, the low reactivity of antibodies showed by CUR patients in the present work suggests that our method can be useful for clinical cure monitoring. These findings agree with other authors that have also shown that low levels of antibodies after therapy can indicate a successful cure [37–39].

Researchers have aimed to overcome the limitations of ATL serological diagnosis by using alternative antigenic preparations to detect *Leishmania* antibodies [32, 33]. The use of live promastigotes would represent a useful tool to increase specificity, since only external membrane epitopes are accessible for IgG binding, avoiding cross reactivity with intracellular components commonly distributed among trypanosomatids [40, 41]. On the other hand, the use of fixed promastigotes represents a practical way to store a massive amount of antigenic support that contributes to a large-scale production. Moreover, the risk of infection during the manipulation of live parasites supports the use of fixed preparations as an antigen source for serological tests. Bittencourt et al., 1968 suggested the possibility of antibody search as a way to monitor ATL cure [42]. In 2001, Brito and colleagues observed increased levels of IgG in spontaneously cured patients, using immunoblot analysis with antigens from soluble and insoluble fractions of the parasite, suggesting an interest in evaluating whether the dynamics of the antibody response could be useful to monitor clinical cure of ATL or not. However, Pereira and colleagues have demonstrated that flow cytometry was not able to detect changes in the anti-*Leishmania* IgG reactivity. A novel methodological approach by delta-reactivity pattern represents a plausible method for post-therapeutic monitoring of ATL at early stages (1 month - 24 months) and after the end of etiological treatment assessment [43]. The proposed strategy analyzed the differential PPFP reactivity detected by paired samples (delta-reactivity), which represents the difference between PPFP after and before treatment. Therefore, serological tests are very important alternatives for monitoring clinical cure, especially because spontaneously cured patients present a decrease in the parasite load and in IgG antibody reactivity [9] and can be identified by flow cytometry technique.

Comparing the performances and accuracies of flow cytometry and ELISA, flow cytometry was shown to be more sensitive and specific as it is observed through the analysis of the ROC curves, showing that if this test is validated, it can be a potential candidate to be used in the laboratorial routine. In fact, this tool can be applicable for cure monitoring of ATL when performed after the end of the etiological treatment. This study demonstrated that flow cytometry was able to distinguish IgG reactivity in patients with active ATL from those who had 1 to 5 years of the end of their treatment. Rocha and Gontijo et al., 2002 reported a reactivity of 21.5% for IFI in individuals without clinical manifestations of ATL who lived in endemic areas for the disease. Although cutaneous lesions caused by *L. (V.) braziliensis* are usually susceptible to antimonial treatment and complete healing occurs at the end of the therapy, the occurrence of relapses strengthens the suggestion that when anti-*Leishmania* antibodies after treatment persist, it could indicate the presence of the parasite and be a predictive factor in ATL’s recurrence [43]. Therefore, we attribute the false-positives 2 years after treatment on flow cytometry to the possibility of reinfection since they live in endemic areas and are immunologically sensitized [36]. Nevertheless, the possibility of cross reactions with other trypanosomatids cannot yet be discarded. Silva et al., 2019 showed that highly specific techniques have to adjust their methods in order to improve their performances as tools for diagnostic confirmation. Regarding the applicability evaluation of IgG1, it was observed that even with the need of a higher number of samples, this approach opens perspectives for its use as a diagnostic tool, as well as for the cure criterion of ATL.

Variables in the quantitative determination of fluorescence intensity were already mentioned, being necessary a careful attention to them during the design phase of the technique’s standardization. These variables involve instruments, antibody (specificity, affinity), samples (type, interfering medications and processing), standardization of the method, analysis and interpretation [44]. However, flow cytometry is one of the most powerful approaches to analyze several kinds of samples in a short period of time, which gives valuable information about the question of interest [23]. Flow cytometry has enabled many assay possibilities, including the serological diagnosis of ATL. It can also be scaled up by using microtiter plates and since it has a high throughput apparatus, it allows the diagnosis of several patients simultaneously [45].

Over the years, many efforts have been made to develop flow cytometers in order to reduce their cost, size and complexity and also to increase their sensitivity [46]. Researchers have reported different flow cytometry platforms, such as an integrated HyperCyt platform on CyAn (Beckman Q3 Coulter), FacScan, and Accuri C6 (BD Bioscience) flow cytometers, as well as the commercial iQue Screener (Intellicyt Corporation, Albuquerque, NM, USA) [45, 47].

## Conclusions

In summary, the performance observed by flow cytometry in the present study strongly reinforces the possibility of using it as an alternative diagnosis for ATL. Through the analyses of the ROC curves, we verified that flow cytometry was superior to ELISA, a test which is used on the laboratorial routine for ATL. We highlight also that there is a possibility of using this methodology as a cure criterion since it was possible to distinguish patients with the active disease from those who were cured. Also, with the data obtained until now, we suggest that the IgG1 isotype can also be applied to ATL’s diagnosis since it was positive in patients with the active disease. Overall, our data suggest the potential and applicability of flow cytometry-based methodologies as an alternative serological diagnosis for ATL. These results open up new avenues of research using flow cytometry, such as understanding the humoral response in the active infection and in patients cure monitoring.

## Data Availability

We affirm that the all data and materials which were generated and analyzed during this study are included in this published article. And also, that the datasets used and analyzed during the current study are available from the corresponding author on request.

## Abbreviations

ATL: American Tegumentary Leishmaniasis
CUR: Subjects spontaneously cured ATL
ELISA: Enzyme-linked immunosorbent assay
NI: Non-infected controls
PCR: Polymerase chain reaction
PPFP: Percentage of positive fluorescent parasites
